# Beyond the visible lesion: mycotoxin attenuation and spatial heterogeneity in maize cobs

**DOI:** 10.1007/s12550-026-00668-8

**Published:** 2026-08-11

**Authors:** Bertha Kachala, Limbikani Matumba, Lindy J. Rose

**Affiliations:** 1 FoodPlus research group, Faculty of Life Science and Natural Resources, Lilongwe University of Agriculture and Natural Resources (LUANAR), Box 143, Lilongwe, Malawi; 2 Department of Plant Pathology, Stellenbosch University, Private Bag X1, Matieland, Capetown, 7602 South Africa

**Keywords:** pre-shelling sorting, Cob-level segregation, Partial salvage, Distance-response modelling, *Fusarium*, Postharvest risk management, Food-safety control

## Abstract

Visible mould is widely used as a practical sorting cue in maize, but it remains unclear whether the visible lesion marks the boundary of mycotoxin contamination within an intact cob. This study examined mycotoxin attenuation, spatial heterogeneity, and co-occurrence in visibly mouldy white maize cobs, using measured distances from the visible mould margin. Cobs with visible mould (*n* = 35) were sampled for the study. For each cob, the visible mould margin was coded as 0 mm, and adjacent sections were collected at the 50% and 75% points along the measured distance from the lesion boundary to the distal cob tip, resulting in 98 cob-section observations. Mycotoxins, FUMs (FB1, FB2, FB3), deoxynivalenol (DON), zearalenone (ZEA), nivalenol (NIV) and AF (AFB1) were quantified by liquid chromatography tandem mass spectrometry. Samples with no detectable levels of mycotoxins were replaced with zero, and total FUMs were calculated (FB1 + FB2 + FB3). The visible mould margin accounted for the largest share of the measured toxin burden, accounting for 84.4% of total FUM, 89.4% of DON, 77.8% of ZEA, and 92.2% of NIV. Adjacent sections retained measurable residual mycotoxins, including 15.6% of total FUM, 10.6% of DON, 22.2% of ZEA, and 7.8% of NIV. In full-distance models, DON, ZEA, and NIV declined significantly with distance, whereas total FUM did not show significant monotonic attenuation. The AFB1 was detected in only three cob sections; these detections co-occurred with *Fusarium*-associated toxins. Co-occurrence analysis showed that adjacent sections frequently remained positive for multiple *Fusarium*-associated toxin groups, with the FUM + DON + ZEA profile persisting across measured-distance bands. These findings show that visible mould is a useful warning signal but not a reliable toxicological boundary. The results support cob-level rejection or segregation of visibly mouldy cobs before shelling, rather than lesion-level trimming, as a more defensible food-safety control measure.

**Supplementary Information:**

The online version contains supplementary material available at https://doi.org/10.1007/s12550-026-00668-8.

## Highlights

Cob sections adjacent to visible mould retained co-occurring mycotoxins.

Mycotoxins DON, ZEA and NIV attenuated more clearly than fumonisin (FUM).

Mouldy cobs should be rejected before shelling.

**Supplementary Information:**

The online version contains supplementary material available at https://doi.org/10.1007/s12550-026-00668-8.

## Introduction

Mycotoxin contamination in maize remains a major food-safety control challenge because the hazard is invisible, heterogeneously distributed and difficult to manage in food systems with limited analytical testing. Important mycotoxin groups in maize include fumonisins (FUM), deoxynivalenol (DON), zearalenone (ZEA), nivalenol (NIV) and aflatoxins (AF). Fumonisins are mainly associated with FUM-producing *Fusarium* species, particularly *F*. *verticillioides* and *F*. *proliferatum* (Bryła et al. 2022). Deoxynivalenol, NIV and ZEA are commonly associated with trichothecene- and ZEA-producing *Fusarium* species, including species within the *F*. *graminearum* species complex (Bryła et al. 2022). Aflatoxins are biologically distinct from the dominant *Fusarium*-associated toxin groups because they are mainly associated with *Aspergillus* species (Palumbo et al. 2020).

Grain sorting is one of the most practical postharvest control measures for reducing mycotoxin exposure in maize. Experimental maize studies show that hand sorting, washing, and dehulling can reduce AF and FUM contamination, with hand sorting producing the greatest reduction (Matumba et al. 2015). Household-level evidence from Tanzania further shows that locally feasible postharvest practices, including hand sorting of maize cobs and grains before storage and use, can reduce AF and FUM contamination and lower subsequent dietary exposure among infants (Kamala et al. 2018). More recent low-cost and technology-assisted sorting studies show that physical, visual and spectral kernel features can be used to remove high-risk maize fractions (Pascale et al. 2020; Aoun et al. 2020; Stafstrom et al. 2021; Mohamed et al. 2023; Chavez et al. 2023). Together, these studies establish sorting as a practical mycotoxin-control intervention, but they do not resolve the cob-level spatial question: once visible mould is observed, can the mouldy portion be removed while retaining neighbouring, visually acceptable tissue, or should the cob itself be treated as the unit of risk?

This question is biologically plausible because maize cobs can support multiple routes of fungal entry, invisible colonisation and co-occurring Fusarium species and mycotoxins (Palumbo et al. 2020; Sherif et al. 2023). It is also practically important because cob-level decisions often occur before shelling, when visually compromised cobs can still be separated before their kernels are mixed into a larger grain lot. However, measured-distance evidence linking visible mould, neighbouring cob tissue and multi-mycotoxin contamination remains limited.

This study examined the spatial distribution and co-occurrence of mycotoxins in visibly mouldy white maize cobs using measured distance from the visible mould margin. The primary objective was to determine whether visible mould corresponds to a localised contamination zone or indicates a broader cob-level mycotoxin-control concern. The secondary objective was to determine whether different toxin groups show similar or divergent spatial patterns away from the visible mould margin. We tested three hypotheses: firstly, that the visibly mouldy point carries the highest mean mycotoxin burden; secondly, that adjacent, apparently clean sections retain measurable residual toxin burden; and thirdly, that mycotoxins differ in their distance-response and co-occurrence patterns. By addressing these hypotheses, the study provides evidence for refining cob-level sorting guidance as an early postharvest food-safety control measure before shelling and mixing.

## Materials and methods

### Study material and sampling design

**Fig. 1:**
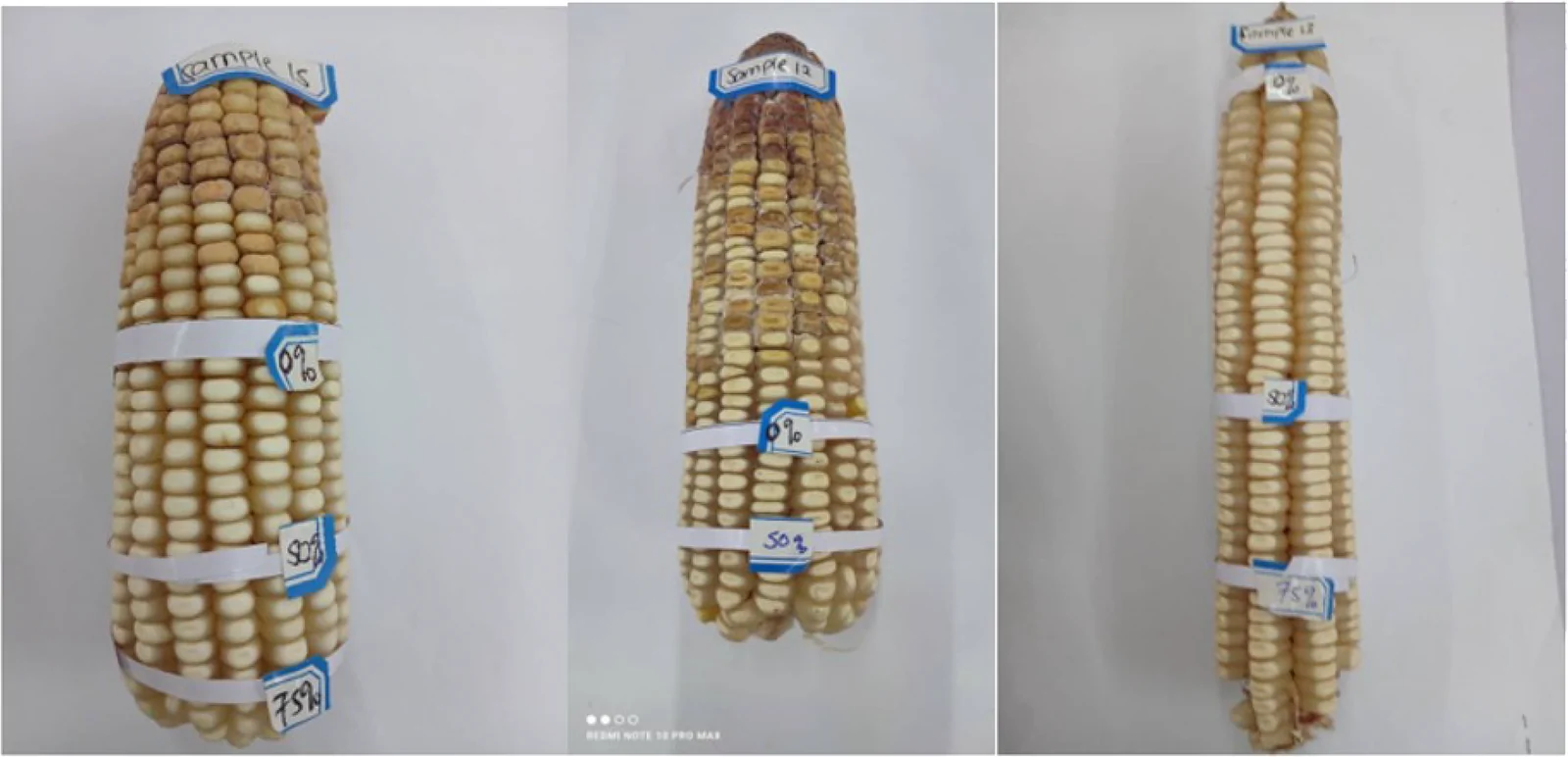
Representative visibly mouldy white maize cobs and cob-section sampling scheme. The visible mould margin was coded as 0 mm, and adjacent sampling points were defined along the measured distance from the lesion boundary to the distal cob tip at 50% and 75% of that distance. The figure illustrates the cob-level sampling layout used before section-specific mycotoxin analysis

The study used visibly mouldy white maize cobs collected from smallholder farmer fields in Lilongwe district. The cobs represented a composite sample, and cultivar identity was not recorded. Intact cobs were selected when they had a clearly visible mould lesion that could serve as a spatial reference point for sampling. Other visible ear rot-associated features, including starburst-like symptoms and white streaking on kernels, were noted where present, but they were not used as inclusion criteria, diagnostic categories or analytical grouping variables. Mould type was also not formally characterised. The visible mould lesion was therefore used only as the reference point for spatial sampling and was coded as 0 mm. Adjacent sampling was based on the measured length from the edge of the visible lesion to the distal cob tip in the direction of sampling. The first adjacent section was taken at the midpoint of this measured length (50%), and the second adjacent section was taken at three-quarters of the same length (75%). In a few short cobs, the 75% section could not be sampled practically without insufficient material or overlap with the cob end; this accounted for the lower number of second-adjacent observations. The final analytical dataset contained 98 cob-section observations from 35 white maize cobs. All 35 cobs contributed one observation from the visible mould margin and one 50% adjacent observation, while 28 cobs contributed an additional 75% adjacent observation. Representative images of the cobs and the sampling scheme are in Fig. 1.

During sampling, distances from the visible mould margin were measured, together with the end-of-mould position, cob length, and cob circumference. Cob length was calculated from the end-of-mould measurement and the lesion-boundary-to-tip distance, with the lesion-boundary-to-tip distance derived from the 50% adjacent-section distance. Cob circumference was used as the measured descriptor of cob size. The cob-section sampling geometry and adjacent-section detection status are summarised in Table 1, while adjacent-section exceedance and cob-morphology association results are reported in Supplementary Tables 1 and 2.

**Table 1:** Cob-section sampling geometry and adjacent-section mycotoxin detection in visibly mouldy maize cobs

Variable	Visible mould margin / 0%	50% adjacent section	75% adjacent section
Number of observations	35	35	28
Section definition	Visible mould margin / reference section	0.50 x lesion-boundary-to-tip distance	0.75 x lesion-boundary-to-tip distance
Distance from visible mould margin, mm	0	58.5 +/- 19.7 range 23.0–95.0	95.9 +/- 26.6 range 43.8-142.5
Detectable adjacent mycotoxins	Reference section	FUM: 35/35 (100.0%) DON: 32/35 (91.4%) ZEA: 28/35 (80.0%) NIV: 9/35 (25.7%) AFB1: 1/35 (2.9%)	FUM: 27/28 (96.4%) DON: 24/28 (85.7%) ZEA: 20/28 (71.4%) NIV: 6/28 (21.4%) AFB1: 0/28 (0.0%)

FUM = total fumonisins, calculated as FB1 + FB2 + FB3; DON = deoxynivalenol; ZEA = zearalenone; NIV = nivalenol; AFB1 = aflatoxin B1. Cob length was 174.6 +/- 32.3 mm (range 124.9–230.0 mm), and cob circumference was 78.6 +/- 59.9 mm (range 15.0–181.0 mm). Missing or non-detect analytical mycotoxin values were treated as zero before analysis. Section mass and direct visibly mouldy-section size were not recorded; therefore, cob length, cob circumference and sampling distance are reported as available cob-morphology and sampling-geometry descriptors rather than direct measures of lesion size or lesion mass

### Sample preparation

After visual marking, each intact cob was divided into the visible mould section and the adjacent sections defined along the sampling direction from the lesion boundary to the distal cob tip (Fig. 1). For each marked section, all kernels assigned to that section were removed and pooled as one cob-section sample. The cob rachis was excluded from the analytical sample. Sectioning and kernel removal were conducted using a clean knife, that was cleaned between sections and cobs to minimise cross-contamination. Each pooled section sample was milled to a fine meal using a laboratory blender and thoroughly mixed before the 5 g analytical subsample was weighed for extraction.

### Mycotoxin analysis and analytical variables

Mycotoxin analysis was performed using liquid chromatography-tandem mass spectrometry (LC-MS/MS). Sample preparation and mycotoxin extraction were conducted following the LC-MS/MS method described by Meyer et al. (2019), with minor modifications. A 5 g of finely milled maize sample was weighed into a 50 mL Falcon tube. Mycotoxins were extracted by adding 20 mL of extraction solution consisting of H₂O: MeOH: AcCN at a ratio of 50:25:25 (v/v/v). The samples were vortexed for 1 min to ensure proper mixing and then incubated in a horizontal position on a shaker-incubator for 30 min, at 260 rpm at room temperature.

After extraction, the samples were centrifuged at 3,000 rpm for 10 min at room temperature. A 5 mL aliquot of the extract was then transferred into a 15 mL Falcon tube and diluted with 5 mL of dilution solution consisting of 25% MeOH in H₂O (v/v). Thereafter, 2 mL of the diluted extract was filtered through a nylon syringe filter directly into LC-MS/MS vials. Multi-mycotoxin analysis was performed using liquid chromatography–tandem mass spectrometry (LC-MS/MS) for fumonisin B₁ (FB₁), fumonisin B₂ (FB₂), fumonisin B₃ (FB₃), deoxynivalenol, zearalenone, nivalenol, and AFB₁. Total FUM was calculated for each sampled section as the sum of FB1, FB2, and FB3 with concentrations expressed as µg/kg. The co-occurrence analysis focused on the four dominant Fusarium-associated toxin groups: FUM, DON, ZEA and NIV. AFB1 was retained in concentration summaries but excluded from the main co-occurrence framing because it occurred only sporadically.

The recoveries were 72–94% for FB₁, 63–90% for FB₂, 68–87% for FB₃, 80–93% for DON, 72–83% for ZEA, and 76–106% for AFB₁ (Meyer et al. 2019). The LOQs were 0.02 mg/kg for FB₁, FB₂, FB₃, and ZEA, 0.1 mg/kg for DON, and 0.002 mg/kg for AFB₁; NIV was assigned a reporting limit of 0.1 mg/kg.

### Data treatment

Missing or non-detect analytical mycotoxin values were treated as zero before analysis. Zero imputation allowed all observations to contribute to mean concentration summaries, burden partitioning, paired comparisons, co-occurrence profiles and distance models. Zero-imputed means were used as the primary concentration metric because the aim was to quantify section-level average burden, including non-quantified results.

### Spatial variables

Spatial position was analysed using three complementary definitions. Sampling-position analysis compared the visible mould margin with the 50% and 75% adjacent sections. Measured distance bands grouped observations into 0 mm, > 0–50 mm, > 50–75 mm, > 75–100 mm, and > 100 mm. Continuous distance used the measured millimetre distance from the visible lesion as the predictor in distance-response models.

### Data analysis

Data analysis was conducted to characterise toxin burden, spatial attenuation, within-cob residual contamination, and co-occurrence patterns. For each toxin, the share of total measured burden at each sampling position was calculated by dividing the summed mycotoxin concentration at that position by the summed concentration across all sampled positions. Adjacent residual burden was calculated as the combined share of the first and second adjacent sections. The sampling design generated section-specific concentration data; therefore, burden shares were expressed on a concentration basis.

Within-cob retention was calculated by comparing each adjacent-section concentration with the corresponding visible mould concentration from the same cob and expressing the adjacent value as a percentage of the visible mould value. Adjacent exceedance was recorded when any adjacent section had a higher concentration than the corresponding visible mould margin.

Distance-response models were fitted using log10(concentration + 1) as the outcome variable and measured distance in millimetres as the predictor. Model coefficients were converted into estimated percentage change per 10 mm increase in distance. Models were fitted first using all observations and then using adjacent sections only after excluding the 0 mm visible mould margin.

Same-section co-occurrence was assessed by counting the number of dominant Fusarium-associated toxin groups present in each sampled section. The co-occurrence analysis focused on FUM, DON, ZEA and NIV. Profiles included any Fusarium-associated toxin group, two or more toxin groups, three or more toxin groups, FUM + DON, FUM + ZEA, DON + ZEA, FUM + DON + ZEA, and FUM + DON + ZEA + NIV. Cob-level co-occurrence was assessed by identifying whether each cob contained specified toxin profiles across any of its sampled sections. Pairwise toxin associations were assessed using Spearman rank correlations on zero-imputed concentrations.

Position-based statistical comparisons were performed on zero-imputed log10(concentration + 1)-transformed values using one-way ANOVA, with sampling position (0%, 50% and 75%) as the grouping factor. Tukey’s honestly significant difference (HSD) test was then used to assign compact letter groupings for pairwise differences among positions.

Associations between cob morphology and mycotoxin outcomes were assessed exploratorily. Spearman rank correlations were used to examine associations between cob length, cob circumference and continuous toxin concentrations at the visible mould margin and in summed adjacent sections. Mann-Whitney tests were used to compare cob length and cob circumference between cobs with and without adjacent-section exceedance. These analyses were interpreted cautiously because cob length and circumference are cob-morphology descriptors rather than direct measurements of visibly mouldy-section size.

Normality of residuals after log10(concentration + 1) transformation was assessed using Q-Q plots and the Shapiro-Wilk test before interpreting the ANOVA outputs; where residual normality remained imperfect, the direction and magnitude of position effects were checked against non-parametric sensitivity analyses.

Data cleaning, statistical analysis and figure generation were conducted in R statistical software. Data management was performed using readxl, dplyr and tidyr, figures were generated using ggplot2, and distance-response models were fitted using the base R lm() function. Concentration data were modelled as log10(concentration + 1), and model coefficients were converted to percentage change per 10 mm increase in measured distance.

## Results

### Toxin burden at the visible mould margin and adjacent sections

The visible mould margin grain fraction had the highest mean concentration for all major toxin groups evaluated, and adjacent sections retained measurable mycotoxin levels (Supplementary Fig. 2). The visible mould margin accounted for 84.4% of total FUM, 89.4% of DON, 77.8% of ZEA and 92.2% of NIV, while adjacent sections accounted for 15.6% of FUM, 10.6% of DON, 22.2% of ZEA and 7.8% of NIV, respectively. AFB1 was detected in only 3 of 98 cob sections and was, therefore, not a dominant contributor to the spatial patterns. Two AFB1 detections occurred at the visible mould margin and one occurred in an adjacent section. All AFB1-positive sections also contained FUM and ZEA, and two also contained DON.

At least one adjacent section contained detectable total FUM and DON in all 35 cobs, while ZEA was detected in adjacent sections of 32/35 cobs, NIV in 12/35 cobs and AFB1 in 1/35 cobs (Table 1). Because adjacent detection was universal or near-universal for the major toxin groups, detection status alone had limited discriminatory value for association testing.

Exploratory Spearman analyses showed toxin-specific relationships between cob morphology and mycotoxin concentrations, but no simple or consistent pattern indicating that residual adjacent contamination was explained by cob length or cob circumference (Supplementary Table 2). Cob length was positively associated with total FUM at the visible mould margin (rho = 0.654, *p* < 0.001), whereas its association with summed adjacent total FUM was weaker (rho = 0.289, *p* = 0.092). Cob length was negatively associated with summed adjacent DON (rho = -0.349, *p* = 0.040) and summed adjacent ZEA (rho = -0.418, *p* = 0.012), while NIV did not show a clear concentration association with cob length. Cob circumference was negatively associated with summed adjacent total FUM (rho = -0.625, *p* < 0.001), but showed no clear association with DON, ZEA or NIV. Adjacent-section exceedance analyses were exploratory and did not show a consistent relationship with cob length or circumference across toxin groups.

The visible mould margin had the highest mean concentration for the main toxin groups (Table 2). ANOVA indicated significant overall position effects for FB1, FB2, FB3, total FUM, DON, ZEA and NIV, but not for AFB1. There was no significant difference in toxin concentrations between the 50% and 75% adjacent positions.

**Table 2:** Position-specific mycotoxin concentrations at the visible mould margin (0%) and adjacent cob Sect.  (50% and 75%) reported as mean +/- SD (mg/kg) with Tukey HSD comparisons

Toxin group	Total FUM	DON	ZEA	NIV	AFB1
0% mean ± SD	39.2 ± 73.5a	11.3 ± 14.4a	5.18 ± 8.32a	0.316 ± 1.15a	0.059 ± 0.318a
50% mean ± SD	3.69 ± 7.16b	0.818 ± 2.29b	1.06 ± 2.52b	0.0248 ± 0.138b	0.000 ± 0.000a
75% mean ± SD	4.46 ± 10.1ab	0.648 ± 1.85b	0.525 ± 1.04b	0.0023 ± 0.0069b	0.000 ± 0.000a

Concentrations are presented as mean +/- standard deviation in mg/kg. The 0% and 50% positions each included 35 observations, while the 75% position included 28 observations. ANOVA and Tukey HSD tests were performed on zero-imputed log10(concentration + 1)-transformed values, with sampling position as the grouping factor. Within each toxin column, values sharing at least one superscript letter are not statistically different (Tukey HSD, p > = 0.05), whereas values with no letter in common differ significantly (*p* < 0.05)

**Fig. 2:**
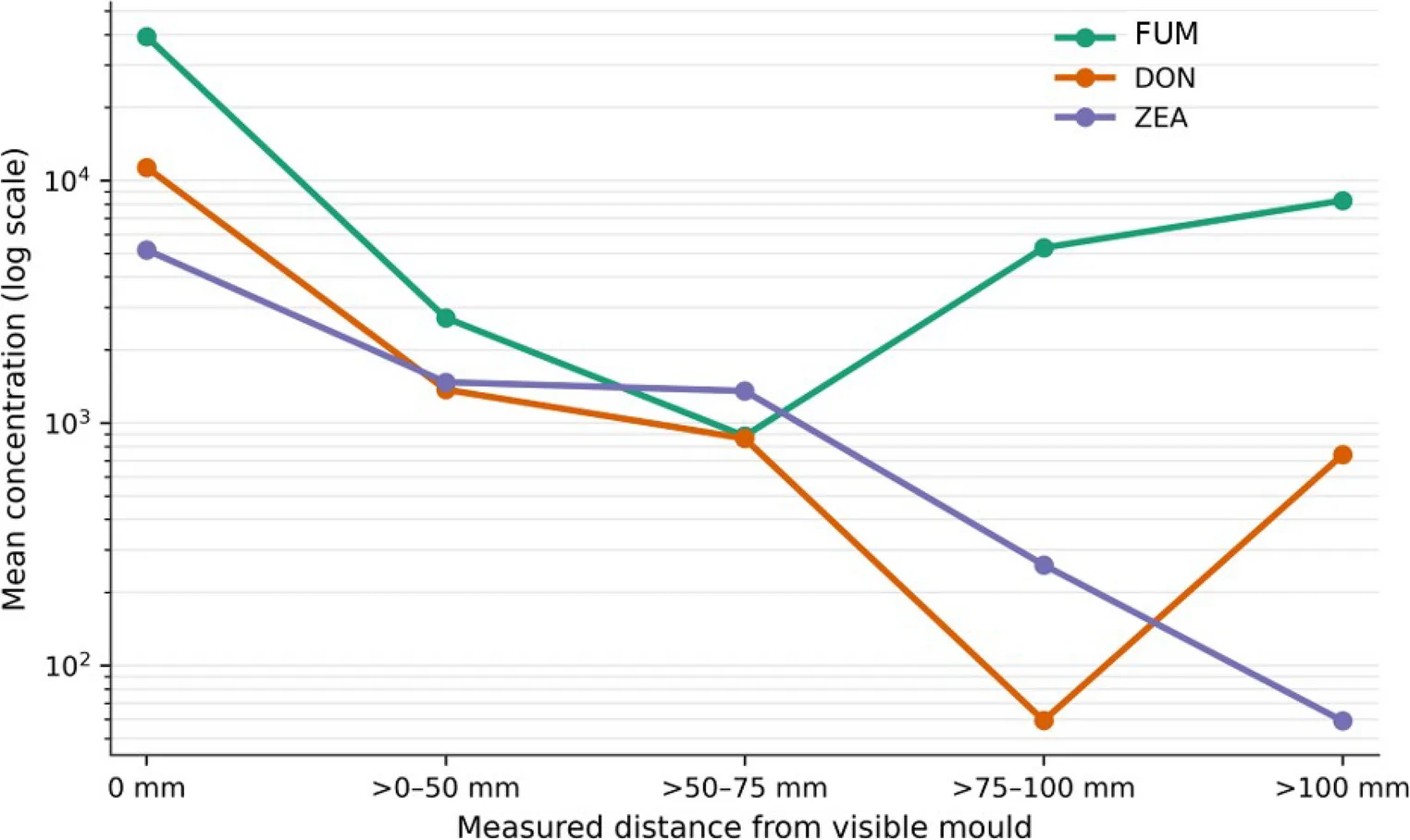
Mean concentrations of total fumonisins (FUM), deoxynivalenol (DON) and zearalenone (ZEA) at different measured distances from the visible mould margin on a maize cob. Values are zero-imputed means. The y-axis is displayed on a log scale to show trends across toxins with different concentration ranges

Measured-distance profiles showed toxin-specific patterns across distance bands (Fig. 2). DON and ZEA generally had lower mean concentrations beyond the visible mould margin, whereas total FUM showed a non-monotonic pattern, with some farther adjacent sections having higher concentrations than nearer sections.

### Distance-response models

Distance-response models showed toxin-specific associations with measured distance from the visible mould margin (Table 3). In the full-distance model, DON, ZEA and NIV had significant negative distance coefficients, with estimated reductions of 34.0%, 26.9% and 13.0% per 10 mm, respectively. Total FUM did not show a significant monotonic (consistent distance-trend) association in the full-distance model.

In the adjacent-section model, DON and ZEA retained significant negative distance coefficients, with estimated reductions of 31.6% and 33.5% per 10 mm, respectively. Total FUM had a significant positive fitted coefficient of 30.7% per 10 mm, while NIV showed no significant adjacent-section distance association.

**Table 3:** Distance-response model estimates for total fumonisins (FUM), deoxynivalenol (DON), zearalenone (ZEA) and nivalenol (NIV) concentration as a function of the measured distance from the visible mould margin

Model	Toxin group	*N*	Estimated change per 10 mm (%)	95% CI (%)	*p*-value
Full-distance model	Total FUM	98	-5.8	-17.7 to 7.8	0.381
Full-distance model	DON	98	-34.0	-42.2 to -24.6	< 0.001
Full-distance model	ZEA	98	-26.9	-36.1 to -16.2	< 0.001
Full-distance model	NIV	98	-13.0	-20.1 to -5.3	0.002
Adjacent-section model	Total FUM	63	30.7	7.2 to 59.3	0.009
Adjacent-section model	DON	63	-31.6	-44.4 to -15.9	< 0.001
Adjacent-section model	ZEA	63	-33.5	-46.9 to -16.7	< 0.001
Adjacent-section model	NIV	63	1.5	-8.3 to 12.4	0.770

Models assessed whether measured distance from the visible mould lesion predicted used log10(concentration + 1) transformed mycotoxin concentration. Estimated changes are expressed per 10 mm increase in measured distance from the visible mould margin. The full-distance model included the visible mould margin and all adjacent sections. The adjacent-section model included only sections outside the visible mould margin

### Within-cob retention and adjacent exceedance

Within-cob comparisons showed that adjacent sections contained measurable mycotoxins for all toxin groups (supplementary Table 1) investigated, and in some cobs the 50% or 75% adjacent section contained higher concentrations than the visible mould section .

### Fusarium-associated multi-toxin co-occurrence

**Fig. 3:**
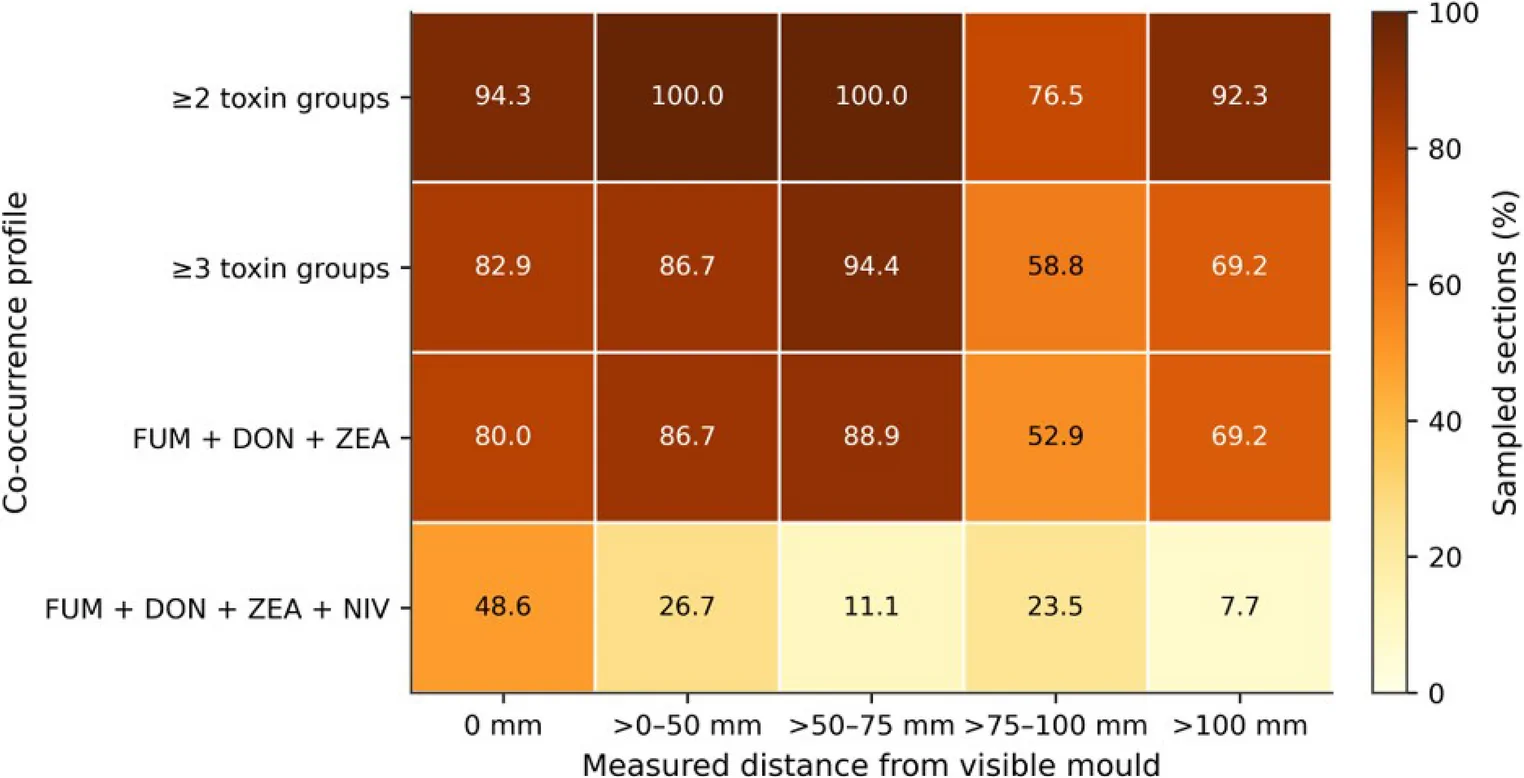
Heatmap of same-section co-occurrence of Fusarium-associated mycotoxins across measured-distance bands. Cell values show the percentage of sampled cob sections within each distance band containing the specified co-occurrence profile. FUM = fumonisins; DON = deoxynivalenol; ZEA = zearalenone; NIV = nivalenol

Co-occurrence analysis focused on FUM, DON, ZEA and NIV. Adjacent sections frequently remained positive for multiple Fusarium-associated toxin groups, and the FUM + DON + ZEA profile persisted across measured-distance bands (Fig. 3).

At cob level, all cobs evaluated contained at least two Fusarium-associated toxin groups. The FUM + DON + ZEA profile occurred in 34 of 35 cobs, and the FUM + DON + ZEA + NIV profile occurred in 62.9% of cobs.

## Discussion

### From sorting efficacy to spatial control

The main contribution of this study is to move cob sorting from a general visual rejection practice toward a spatially informed food-control decision. Sorting is already supported by experimental, household and technology-assisted evidence, including hand sorting, washing, dehulling, cleaning, low-cost kernel separation and spectral sorting approaches that reduce mycotoxin burden by removing high-risk maize fractions (Matumba et al. 2015; Kamala et al. 2018; Aoun et al. 2020; Pascale et al. 2020; Stafstrom et al. 2021; Chavez et al. 2023; Mohamed et al. 2023). The present study does not contradict this evidence; rather, it addresses how visible symptoms should be interpreted while the maize grain is still on the cob. By linking measured distance from visible mould with multi-mycotoxin distribution, this study shifts the sorting decision to the pre-shelling stage, when the biological unit of concern is still identifiable and can be segregated before its kernels are mixed into the grain stream.

### Partial salvage, distance evidence and the limits of visual boundaries

The lack of a significant difference in toxin concentration between the 50% and 75% positions does not necessarily mean that the two adjacent fractions were biologically equivalent, because those percentage points represented different absolute millimetre distances on short and long cobs. This helps explain why the percentage-position comparison was less spatially discriminating, while the measured-distance analysis more clearly showed the contrast between the less regular FUM pattern and the stronger attenuation of DON, ZEA and NIV.

The decision to remove only the visibly mouldy portion of maize grain is not merely hypothetical. In Mozambique, farmer interviews indicated that although many farmers recognised fungal contamination in maize, a substantial proportion assumed that the mouldy part could be removed and the remaining maize could be safe for consumption (Matusse et al. 2025). Such partial salvage treats the visible mould margin as an informal food-control boundary. That logic would be defensible only if toxin burden declined predictably with distance from the lesion and if neighbouring visually acceptable tissue represented a consistently lower-risk fraction. This assumption is especially weak for fumonisins, because *F. verticillioides* infection is often spatially heterogeneous and may occur without visible symptoms; more broadly, toxigenic fungi can colonise apparently normal kernels without producing clear external signs.

The distance patterns observed in this study weaken that boundary assumption. Although several toxins attenuated away from the visible mould margin, the response was not uniform across mycotoxin groups, and some farther adjacent sections showed higher concentrations than nearer sections. This non-monotonic pattern should not be interpreted as proof of simple toxin movement from one visible source. Instead, it indicates that contamination beyond visible mould may arise through more than one process.

Several biological mechanisms could contribute to this pattern. Maize ear infection can occur through the silk channel, wounds, and kernel infection courts, and major silk-entering pathogens include F. graminearum and F. verticillioides (Thompson and Raizada 2018). Experimental work using F. verticillioides and silk-channel inoculation documented kernel infection courts and early colonisation within maize ears, while F. graminearum has been shown to colonise maize silks (Duncan and Howard 2010; Miller et al. 2007; van Dyk et al. 2021). Fusarium verticillioides can also colonise maize systemically through the roots or stalks with infections being symptomless, supporting the possibility that visually normal tissue may still be biologically compromised (Bacon and Hinton 1996; Murillo-Williams and Munkvold 2008). These colonisation pathways make it plausible that toxin-positive tissue away from visible mould could reflect hidden infection, a separate secondary infection point beyond the lesion selected for sampling, localised toxin production in visually normal kernels, or uneven distribution of contaminated kernels within the same cob.

Chemical differences among toxin groups in the present study provide an additional reason why spatial behaviour may differ. This divergence is biologically plausible because FUM are highly polar and water-soluble mycotoxins with multiple hydroxyl and tricarballylic acid groups, whereas zearalenone is more hydrophobic and estrogenic, and DON and NIV are trichothecenes with different molecular structures and biosynthetic origins (Kamle et al. 2019; Bryła et al. 2022). These differences may influence how each toxin is synthesized in cob tissues depending on moisture availability, associates with kernel matrices, persists after fungal growth, or appears in sampled sections. They may also help explain why toxin groups that co-occur on the same cob do not necessarily show the same distance-response pattern. However, the present study did not measure toxin mobility, tissue binding, fungal biomass, or water movement, so chemistry should be treated as a plausible contributor to spatial heterogeneity rather than evidence of toxin migration.

The observed spatial patterns are, therefore, better interpreted as the combined result of fungal colonisation pathways, toxin biosynthesis, chemical properties and kernel-level heterogeneity. A purely migration-based explanation would be too narrow as toxin-positive tissue, away from visible mould, could arise from endophytic fungal growth, separate infection points, local biosynthesis in visually normal kernels, persistence of preformed toxins, limited local movement, or some combination of these processes. The food-control significance is specific but important as visual distance from mould does not reliably define a safe retained portion of the cob. The visible lesion identifies a contaminated cob, but it does not reliably define the spatial limit of mycotoxin contamination. For this reason, the findings are better framed as evidence of spatial extension and heterogeneity as opposed to evidence of mycotoxin translocation.

### Co-occurrence, fungal interactions and biosynthetic separation

The co-occurrence findings also argue against discussing the data as a single-toxin sorting problem. The persistence of FUM + DON + ZEA profiles across distance bands means that adjacent material cannot be treated as analytically clean simply because one toxin group attenuates more clearly than another. Most of the cobs evaluated contained at least two mycotoxins, and more than half contained up to three mycotoxins. These co-occurrence results should be interpreted as evidence of cob-level fungal ecology rather than simply a catalogue of toxin combinations. Maize ears can support overlapping toxigenic systems, when environmental conditions are favourable. Interactions between *F. verticillioides* and *F. graminearum* in maize ears have been shown to influence fungal development and mycotoxin accumulation (Picot et al. 2012). More recent work also shows that these fungi interact during co-infection, with changes in both fungal growth and toxin production when species co-occur (Sherif et al. 2023). The reported co-occurrence of DON and FUM in maize-based matrices further supports the view that maize safety should not be considered one toxin at a time (Palumbo et al. 2020).

Co-occurrence, however, does not imply uniform spatial behaviour. Fumonisins, trichothecenes like DON and ZEA are associated with different Fusarium toxins and biosynthetic pathways (Bryła et al. 2022). The same cob may, therefore, support several toxigenic systems that overlap spatially but respond differently to microclimate, kernel maturity, insect injury, host tissue, and fungal competition. This distinction is important for interpreting the present findings: a cob may carry a multi-toxin profile even when individual toxin groups attenuate or persist differently with distance. Cob-level sorting is, therefore, a realistic response to biological non-uniformity, not a claim that all toxins share one source or one spatial pathway. The rare AFB1 detections in this study indicate that *Aspergillus*-associated contamination can occur alongside *Fusariu*m-associated mycotoxins, but their low detection frequency in this dataset supports keeping the main co-occurrence interpretation centred on FUM, DON, ZEA and NIV.

### Implications for food-control practice and food-security-sensitive implementation

Because adjacent sections in the present study still retained measurable mycotoxin contamination, visible mould is better interpreted as a cob-level warning signal than as a boundary for trimming. However, cob-level segregation will only be effective if it is implemented within the realities of household food security and smallholder income. A visibly mouldy cob is not only a safety risk but also represents food, feed or cash value. Evidence from Malawi shows that although many people recognize mould as undesirable, knowledge of mould toxins and safe management remains limited, including misunderstandings about toxin persistence after cooking and the continued purchase or use of visibly mouldy maize or other foods (Matumba et al. 2016; Batch et al. 2024). Studies in other African countries have shown limited farmer knowledge of fungal and mycotoxin risks, alongside assumptions that mouldy portions can be removed while the remaining maize is used (Phokane et al. 2019; Bila et al. 2025; Matusse et al. 2025). These findings indicate that cob sorting cannot be treated as a technical instruction alone but requires risk communication that explains why visible mould may signal invisible contamination beyond the lesion.

Rejection messages also need practical and economic support. Without acceptable alternatives, visibly mouldy cobs may be consumed, sold cheaply, or diverted into feed, shifting rather than reducing exposure risk. Regional guidance on AF-contaminated commodities recognises that disposal should be complemented, where feasible, by regulated alternative uses such as controlled non-food, energy or tightly managed feed pathways (EAC, 2017). Cob-level segregation should, therefore, be linked to safe diversion systems, incentives, and value-preserving options that reduce the pressure to salvage unsafe material.

For formal value chains, cob-level segregation can serve as an early barrier within a broader mycotoxin-control system that includes rapid drying, moisture control, storage management, grain cleaning, kernel sorting, and analytical verification. For household systems, the message should remain simple but realistic: visibly mouldy cobs should not be trimmed for food use, and farmers need safe, acceptable routes for rejected material. Because segregation can result in food loss, cob sorting should be framed as a food-security-sensitive control measure rather than a stand-alone visual instruction. More importantly, it emphasises the need for preventive measures, including farmer access to maize varieties with resistance or reduced susceptibility to ear rot (Mukanga et al. 2011).

### Limitations

This study was designed to evaluate spatial patterns around visible mould, not to identify the exact biological mechanisms responsible for contamination beyond the visible lesion. The data cannot distinguish mycotoxin translocation from invisible colonisation, independent infection points, persistence of preformed toxins, or kernel-level hotspots. Missing or non-detect analytical values were treated as zero, which allowed consistent mean-based burden analysis but may have underestimated low-level contamination if some missing values represented concentrations below quantification limits rather than true absence. A further limitation is that section mass and direct visibly mouldy-section size were not recorded. The study therefore could not directly determine whether the physical size, mass or area of the visibly mouldy portion predicted adjacent-section contamination. Cob length and cob circumference were available and were used in exploratory association analysis, but these measurements are cob-morphology descriptors rather than direct lesion-size measurements. In addition, fungal ecology was inferred from toxin profiles and literature-supported biological plausibility rather than from fungal isolation, sequencing, or direct measurement of biosynthetic genes.

The sampling structure captured selected cob sections rather than a complete three-dimensional map of each cob. Therefore, the results should be interpreted as evidence of spatial heterogeneity and residual contamination around visible mould, not as a full reconstruction of toxin movement within cob tissues. Future work should combine measured-distance toxin mapping with mould characterisation, fungal isolation, molecular identification, biosynthetic gene markers, ear rot symptom scoring, insect-damage assessment, husk-cover assessment, moisture profiling, and kernel imaging. Field studies should also quantify toxin reduction, grain loss, economic trade-offs, and behavioural acceptability when visibly mouldy cobs are rejected before shelling.

## Conclusions

The first hypothesis, that the visibly mouldy point carries the highest mean mycotoxin burden, was accepted. The visible mould margin accounted for the largest share of the measured burden for total FUM, DON, ZEA and NIV. The second hypothesis, that adjacent apparently clean sections retain measurable residual toxin burden, was accepted. Adjacent cob sections retained measurable mycotoxin concentrations for the major toxin groups, showing that the visible boundary of mould did not correspond to analytical absence in neighbouring material. The third hypothesis, that toxin groups differ in their distance-response and co-occurrence patterns, was accepted. Mycotoxins, DON, ZEA and NIV showed clearer distance attenuation, whereas FUM showed more irregular spatial behaviour. Co-occurrence analysis, restricted to the dominant *Fusarium*-associated toxins, was dominated by the FUM + DON + ZEA profile.

Visible mould should be used as a cob-level sorting cue, not as a guide for trimming only the visibly affected portion. This supports pre-shelling segregation of visibly mouldy cobs before their kernels are mixed into the grain stream.

## Supplementary Information

Suplementary Material 1. Supplementary Figure 1. Residual share of total fumonisins (FUM), deoxynivalenol (DON), zearalenone (ZEA) and nivalenol (NIV) in adjacent cob sections outside the visible mould point. Values show the combined contribution of the 50% and 75% adjacent sections. Supplementary Table 1. Within-cob adjacent-section retention and higher-concentration indicators for total fumonisins (FUM), deoxynivalenol (DON), zearalenone (ZEA) and nivalenol (NIV). Supplementary Table 2. Exploratory Spearman rank correlations between cob morphology and mycotoxin concentrations.

## Data Availability

The datasets generated and analysed during the current study are available from the corresponding author upon reasonable request.
