# Supplementary material for: Beyond the visible lesion: mycotoxin attenuation and spatial heterogeneity in maize cobs
Source: Mycotoxin Res. 2026 Aug 11;42(4):57. doi: 10.1007/s12550-026-00668-8 (PMC13461790; doi:10.1007/s12550-026-00668-8)
Supplement: Supplementary file 1 [file 12550_2026_668_MOESM1_ESM.pdf]

Supplementary Figure 1. Residual share of total fumonisins (FUM), deoxynivalenol (DON), zearalenone (ZEA) and nivalenol (NIV) in adjacent cob sections outside the visible mould point. Values show the combined contribution of the 50% and 75% adjacent sections

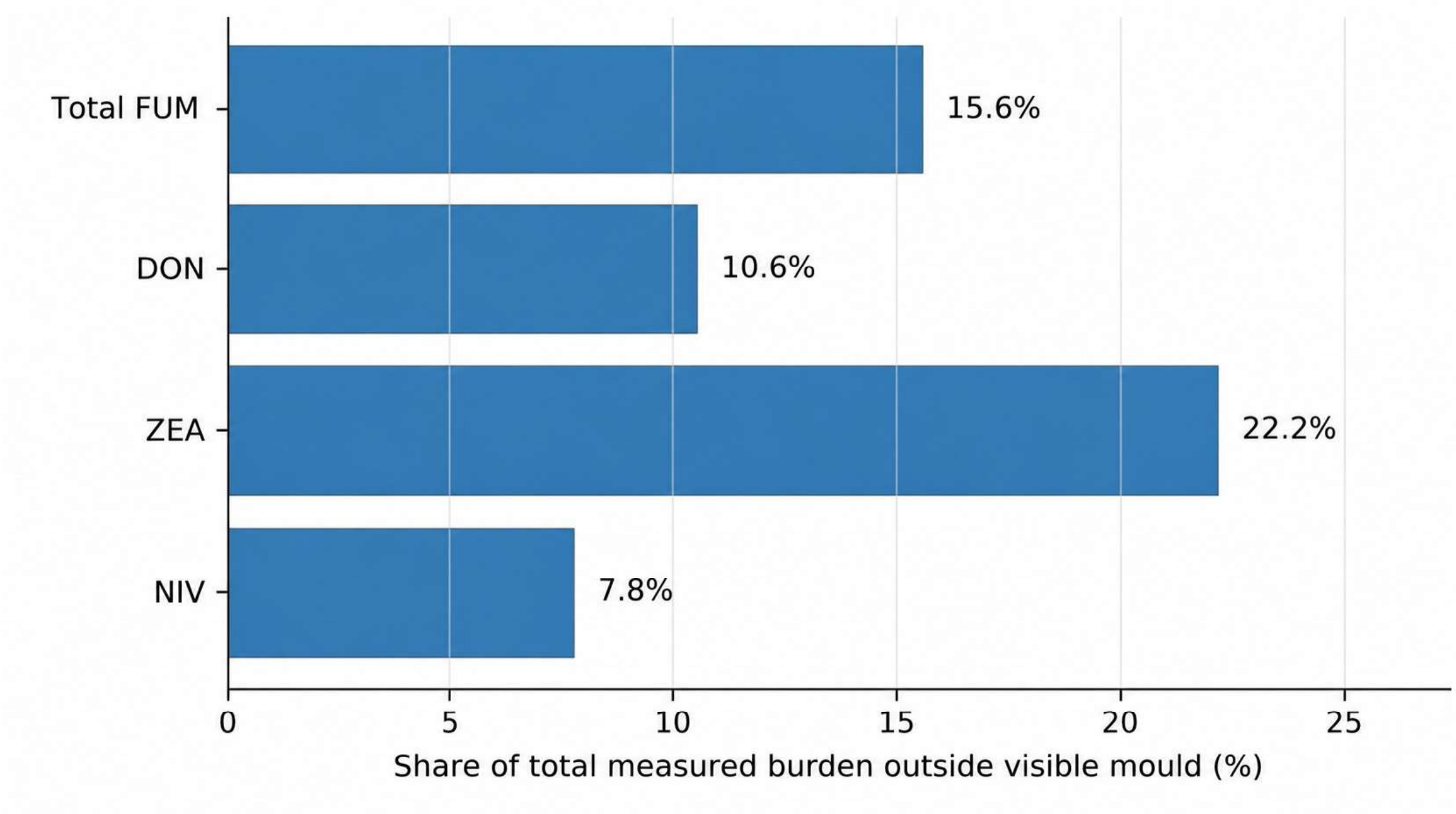

Supplementary Table 1. Within-cob adjacent-section retention and higher-concentration indicators for total fumonisins (FUM), deoxynivalenol (DON), zearalenone (ZEA) and nivalenol (NIV)

Indicator	Total FUMs	DON	ZEA	NIV
First adjacent section retained concentration relative to visible mould section (%)	9.4	7.2	20.4	7.9
Second adjacent section retained concentration relative to visible mould section (%)	10.9	5.7	10.6	0.6
Cobs with at least one adjacent section higher than the visible mould section, n/N (%)	12/35 (34.3)	7/35 (20.0)	10/35 (28.6)	5/35 (14.3)

Note: Retained concentration was calculated as the adjacent-section concentration relative to the corresponding visible mould section from the same cob. A higher adjacent section indicates that at least one adjacent section on that cob had a higher concentration than the visible mould section. FB1 showed this pattern in 14/35 cobs (40.0%)

Supplementary Table 2. Exploratory Spearman rank correlations between cob morphology and mycotoxin concentrations

Cob morphology variable	Toxin group	Visible mould margin, rho	*p*-value	Summed adjacent sections, rho	*p*-value
Cob length	Total FUM	0.654	< 0.001	0.289	0.092
Cob length	DON	-0.338	0.047	-0.349	0.040
Cob length	ZEA	-0.311	0.069	-0.418	0.012
Cob length	NIV	-0.232	0.179	0.167	0.338
Cob circumference	Total FUM	-0.405	0.016	-0.625	< 0.001
Cob circumference	DON	0.057	0.747	0.224	0.195
Cob circumference	ZEA	0.044	0.801	0.032	0.856
Cob circumference	NIV	0.202	0.244	-0.217	0.211

Note: Correlations were exploratory and based on zero-treated analytical mycotoxin values. Summed adjacent sections represent the combined toxin concentration in available adjacent sections for each cob. Section mass and direct visibly mouldy-section size were not measured; therefore, cob length and cob circumference were treated as cob-morphology descriptors rather than direct lesion-size measurements
